# Advances in anti-aging Drug research leveraging multi-omics and artificial intelligence

**DOI:** 10.3389/fragi.2026.1868486

**Published:** 2026-07-30

**Authors:** Lijuan Gao, Yongsheng Qin, Yudai Xu, Ju Su, Zhicheng Cai, Zhongyu Hu, Ruohu Shi, Xinyi Mu, Shiying Cen, Chanchan Xiao, Guobing Chen

**Affiliations:** 1 Department of Microbiology and Immunology, Institute of Geriatric Immunology, School of Medicine, Jinan University, Guangzhou, China; 2 School of Environmental Engineering, Guangdong Polytechnic of Environment Protection Engineering, Foshan, Guangdong, China; 3 The Sixth Affiliated Hospital of Jinan University (Dongguan Eastern Central Hospital), Jinan University, Dongguan, Guangdong, China; 4 First Affiliated Hospital, Jinan University, Guangzhou, Guangdong, China; 5 Office of Scientific Research and Development, Jinan University, Guangzhou, Guangdong, China

**Keywords:** Anti-aging drugs, artificial intelligence, clinical translation, drug screening, multi-omics

## Abstract

The intensifying global population aging has rendered the development of anti-aging drugs a core challenge in the life sciences domain. Traditional models struggle to address the systemic and networked nature of aging. Multi-omics technologies provide a panoramic perspective for deciphering molecular networks of aging, while Artificial Intelligence (AI) has demonstrated significant advantages in target discovery, drug screening, and clinical trial optimization. This review systematically elaborates on the pathological characteristics and molecular mechanisms of aging, analyzes the application paradigms of multi-omics in biomarker screening, mechanism elucidation, and high-throughput screening, and discusses the core value and technical bottlenecks of AI in target prediction, virtual screening, and trial design. Simultaneously, this paper addresses critical challenges including ethical controversies and data standardization currently confronting the field, and explores the prospects of precision anti-aging drug development and personalized treatment strategies driven by deep integration of multi-omics and AI. This approach offers novel theoretical frameworks and practical pathways for extending human healthspan.

## 1 Introduction

The accelerated global population aging represents the most prominent demographic shift of the 21st century, posing substantial challenges to public health systems worldwide and socio-economic development. According to statistics from the World Health Organization (WHO), by 2050, the global population aged 60 and above is projected to exceed 2 billion, accounting for over 22% of the total global population (Wang et al., 2024; Fang et al., 2020). Consequently, the incidence of age-related diseases (such as cardiovascular disease, neurodegenerative diseases, metabolic disorders, and malignant tumors) continues to rise, not only severely affecting the quality of life for the elderly but also imposing a substantial burden on healthcare systems (Gaille et al., 2020). Against this backdrop, slowing aging, extending healthspan, and reducing the risk of age-related diseases have emerged as critical challenges and research priorities in the life sciences field (Lopez Sanchez et al., 2016). Aging constitutes a complex biological process involving multiple dimensions, levels, and pathways. According to the updated framework proposed by López-Otín and colleagues (López-Otín et al., 2023), aging is driven by twelve interconnected hallmarks that fulfill three key criteria: age-associated manifestation, acceleration of aging upon experimental accentuation, and deceleration upon therapeutic intervention. These hallmarks are organized into three categories—primary (genomic instability, telomere attrition, epigenetic alterations, loss of proteostasis, disabled macroautophagy, deregulated nutrient-sensing, mitochondrial dysfunction), antagonistic (cellular senescence), and integrative (stem cell exhaustion, altered intercellular communication, chronic inflammation, dysbiosis). Together, these hallmarks constitute a comprehensive framework for understanding the molecular and cellular basis of aging and for identifying potential therapeutic targets.

Traditional anti-aging drug research predominantly focuses on single targets or signaling pathways, rendering it inadequate to comprehensively address the systemic and networked nature of aging. Consequently, most drug development efforts face the dilemma of “limited efficacy and significant side effects” (Wong et al., 2024; Jiaofen et al., 2024). However, it is important to recognize that currently available anti-aging interventions exhibit distinct and class-specific adverse effect profiles. For example, the mTOR inhibitor rapamycin, while demonstrating lifespan extension in multiple model organisms, is associated with immunosuppression, hyperlipidemia, and insulin resistance in clinical settings (Adav and Wang, 2021). Senolytic agents, such as the Dasatinib + Quercetin combination or the BCL-2/BCL-xL inhibitor Navitoclax, show efficacy in clearing senescent cells but carry risks including hematological toxicity (thrombocytopenia, neutropenia), fatigue, and gastrointestinal disturbances (Skov et al., 2021; Tang et al., 2025). Metformin, widely used for and investigated for geroprotective effects, is generally well-tolerated but can cause gastrointestinal intolerance and, rarely, lactic acidosis (Pietrocola and Kroemer, 2017). Even aspirin, considered for its anti-inflammatory geroprotective potential, demonstrates a dose-dependent increase in bleeding risk (OR = 1.54, 95% CI 1.32–1.80) (Lushchak et al., 2021). These observations underscore that no universal ‘safe’ anti-aging agent exists; instead, risk-benefit assessment must be individualized based on therapeutic class, dosing regimen, patient comorbidities, and frailty status. Consequently, most drug development efforts face the dual challenge of achieving sufficient efficacy while managing predictable, class-specific adverse effects.

In recent years, multi-omics technologies have generated high-dimensional molecular landscapes that systematically define hallmarks of aging, including genomic instability, mitochondrial dysfunction, cellular senescence, chronic inflammation, and dysbiosis (Chen et al., 2024; Solovev, 2024; Long et al., 2025). Meanwhile, artificial intelligence provides the analytical power to integrate heterogeneous omics data, identify core aging signatures, prioritize therapeutic targets, and predict candidate anti-aging compounds (Zagirova et al., 2023; Pun et al., 2023; Liu Y. et al., 2025; Zhang et al., 2025; Galimov and Yakimovich, 2022).

The deep integration of multi-omics and AI establishes a unified data-driven framework that links aging biology to drug discovery, enabling systematic target identification, mechanistic interpretation, high-throughput screening, and precise clinical translation. This synergistic paradigm overcomes the limitations of single-target strategies and facilitates the development of safer and more effective anti-aging interventions (Shao et al., 2024). In 2025, Ren et al. integrated the multi-omics target discovery platform PandaOmics with the generative chemistry platform Chemistry42, developed the TNIK-targeting anti-fibrotic drug INS018_055, and validated its favorable safety and pharmacokinetic profile in a Phase I clinical trial, marking a significant breakthrough in generative AI’s clinical translation for anti-aging drugs (Hofmann, 2023). The pathway- and transcriptome-based deep learning system PTD-DEP identified melatonin, demonstrating its capacity to delay cellular senescence by targeting the P300/BMAL1 axis and significantly improve cognitive function in Alzheimer’s disease model mice (Odenkirk et al., 2021). In virtual screening and senolytic agent discovery, Wong et al. employed a Graph Neural Network to systematically evaluate over 800,000 small molecules, identifying the novel senolytic compound BRD-K56819078, which outperformed traditional Bcl-2 inhibitors in clearing senescent cells in aged mice (Skov et al., 2021). Smer-Barreto et al. utilized an XGBoost Machine Learning Model to screen thousands of compounds, identifying natural products including Ginkgo biloba biflavones and Periplocin that demonstrated significant anti-aging activity (Smer-Barreto et al., 2023). While the synergistic application of multi-omics and AI technologies has shown strong research potential and translational value in drug repositioning, virtual screening, generative molecular design, and preclinical validation, it faces challenges including multi-omics data heterogeneity and standardization issues, AI model interpretability and generalization limitations, the unique requirements of anti-aging drug clinical trials, and the ethical and societal controversies accompanying ‘life extension,’ all requiring systematic examination and thorough investigation (Patel et al., 2020; Wu et al., 2025; Bajenova et al., 2019; Chau et al., 2024).

Aging constitutes a complex pathological process orchestrated by an interconnected multi-pathway network, against which conventional single-target anti-aging therapeutics demonstrate constrained clinical efficacy and notable safety concerns. Multi-omic approaches afford comprehensive characterization of the molecular landscape of aging and facilitate robust biomarker identification. Concurrently, artificial intelligence harnesses high-dimensional omics datasets to enable target mining and *in silico* compound screening. The synergistic integration of these dual modalities underpins the full-spectrum framework for anti-aging drug discovery, therapeutic response assessment, and personalized intervention strategies. Current anti-aging drug research is transitioning from traditional to data-driven paradigms. This review systematically examines aging’s core characteristics and pharmacological intervention strategies, analyzes the implementation pathways, key advantages, and technical limitations of multi-omics and artificial intelligence technologies in target identification, drug screening, and clinical translation, and discusses future trajectories to provide theoretical frameworks and actionable guidance for advancing research in this domain.

## 2 Results

### 2.1 Research strategy

To systematically identify advances in multi-omics and AI-driven anti-aging drug research, we conducted literature searches across PubMed, Web of Science, Google Scholar, Cochrane Library, and EMBASE databases until April 1, 2026. The search terms used were as follows: #1 — “Anti-aging Drugs” or “Anti-aging Pharmacology” or “senolytics”; #2 — “Multi-omics” or “Genomics” or “Transcriptomics” or “Proteomics” or “Metabolomics”; #3 — “Artificial Intelligence” or “Machine Learning” or “Deep Learning” or “Drug Screening”; #4 — “Personalized Treatment” or “Precision Medicine” or “Clinical Translation”; #5 — #1 AND #2; #6 — #1 AND #3; #7 — #1 AND #2 AND #3; #8 — #1 AND #2 AND #3 AND #4.

### 2.2 Inclusion criteria

This review encompasses all literature exploring anti-aging drug research leveraging multi-omics and artificial intelligence. Inclusion criteria specify that only English-language publications—including original research, reviews, case reports, case series, opinion articles, and meta-analyses—explicitly focused on anti-aging drug development are incorporated; Studies must demonstrate applications of multi-omics technologies (e.g., genomics, proteomics, metabolomics) or artificial intelligence methods (e.g., machine learning, deep learning) in anti-aging drug screening, target discovery, or personalized treatment; We also included literature discussing relevant theoretical foundations (e.g., aging biomarker theories, drug-target interaction models) and challenges in technical implementation (e.g., data heterogeneity, model interpretability, clinical translation barriers).

### 2.3 Study selection

**FIGURE 1:**
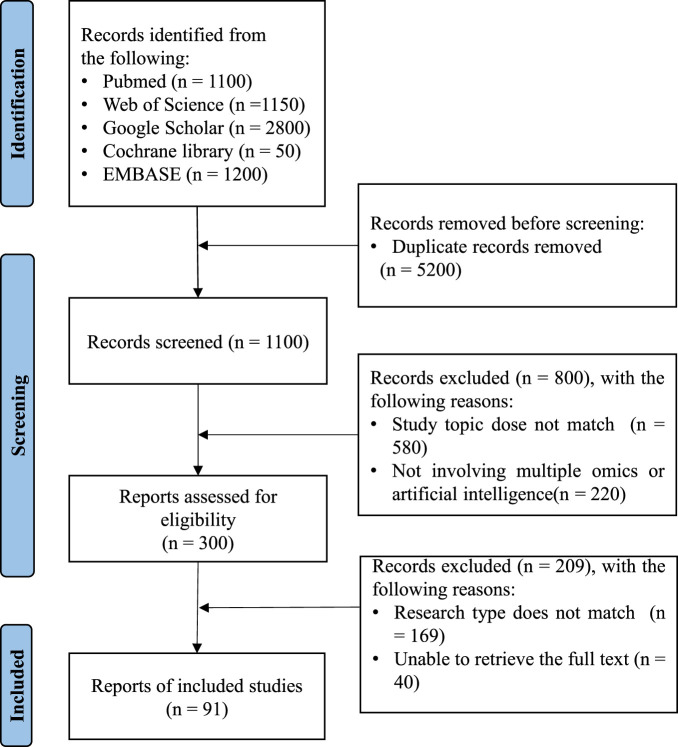
Research selection process of research progress of anti-aging drugs using multi omics and artificial intelligence.

After manually removing duplicate records, we initially screened article titles and abstracts from preliminary searches. Subsequently, full texts of potentially relevant literature underwent detailed review according to inclusion/exclusion criteria (Figure 1). After abstract screening, full texts were assessed for eligibility before final inclusion/exclusion decisions. Any discrepancies in evaluations were resolved through consensus meetings.

## 3 Scientific foundation and core technologies of anti-aging Drug research

### 3.1 Pathological features of aging and intervention strategies

Aging constitutes a multidimensional and progressive pathophysiological process primarily driven by cellular senescence. The conceptual framework of aging hallmarks has evolved from the original nine to twelve, with the recent addition of disabled macroautophagy, chronic inflammation, and dysbiosis—highlighting impaired organelle turnover, sustained low-grade inflammation, and gut microbial dysregulation as core contributors (López-Otín et al., 2023). These novel hallmarks are not isolated but intimately linked to previously recognized ones. Ultimately, these culminate in degenerative aging of tissues and organs and the onset of age-related diseases (Biga et al., 2025).

Among the twelve, six hallmarks—disabled macroautophagy, deregulated nutrient-sensing (mTORC1/AMPK), stem cell exhaustion, altered intercellular communication, chronic inflammation, and dysbiosis—are particularly critical and form a mutually reinforcing network (López-Otín et al., 2023). Disabled macroautophagy impairs clearance of protein aggregates and damaged mitochondria, accelerating proteostasis loss and mitochondrial dysfunction. Deregulated nutrient-sensing becomes pro-aging when hyperactivated in adulthood, whereas moderate inhibition extends lifespan via enhanced autophagy and metabolic adaptation (Saliev and Singh, 2025). Stem cell exhaustion directly reduces tissue regenerative capacity, driving age-related decline. Altered intercellular communication disrupts endocrine, neural, and immune signaling, extending aging effects from cellular to systemic levels. Chronic inflammation (“inflammaging”) serves as a convergence point for damage signals, sustained by the senescence-associated secretory phenotype (SASP), gut-derived mediators, and immune dysregulation (Gaylord et al., 2023). Concurrently, dysbiosis amplifies systemic inflammation by compromising gut barrier integrity and modulates host metabolism/immunity via microbial metabolites (Dhokia et al., 2024). Collectively, these hallmarks culminate in tissue degeneration and age-related diseases (Biga et al., 2025).

**FIGURE 2:**
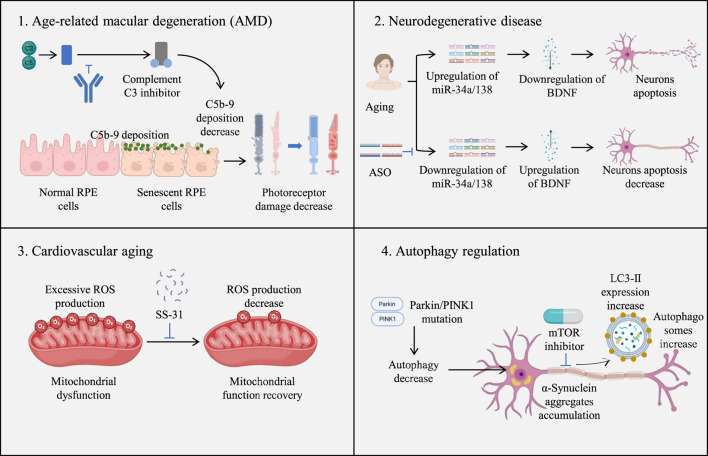
Intervention measures for anti-aging.

Targeted interventions against these core hallmarks have shown therapeutic promise across multiple age-related pathologies. In the visual system, retinal pigment epithelial cell senescence activates complement cascades (C5b-9 deposition) in age-related macular degeneration (AMD); C3 inhibitors targeting this pathway represent a promising intervention (Lyzogubov et al., 2016; Jones et al., 2025). In the nervous system, dysregulated miRNAs (e.g., miR-34a, miR-138) promote neuronal apoptosis by downregulating BDNF, and antisense oligonucleotides targeting these miRNAs exhibit neuroprotective potential (You et al., 2016). Mitochondrial dysfunction drives cardiovascular aging via excessive ROS production; the mitochondria-targeted peptide SS-31 improves mitochondrial function and reduces oxidative damage (Lopez Sanchez et al., 2016). Disabled macroautophagy, a key driver of aging and Parkinson’s disease, is exacerbated by Parkin/PINK1 mutations leading to α-synuclein aggregation; mTOR inhibitors such as rapamycin restore autophagic flux and delay senescence (Ouyang et al., 2016). These examples collectively support that targeting interconnected aging hallmarks—cellular senescence, mitochondrial dysfunction, impaired macroautophagy, dysregulated miRNA signaling, and altered intercellular communication—provides a mechanistically coherent therapeutic strategy for age-related diseases (Figure 2).

The characterization of aging hallmarks and their interconnected molecular networks has not only deepened our fundamental understanding of the biology of aging but has also yielded numerous actionable therapeutic targets. Key pathways—including cellular senescence, chronic inflammation, mitochondrial dysfunction, impaired macroautophagy, and dysregulated nutrient sensing—present promising opportunities for pharmacological intervention. In summary, aging is a pathological process centered on a network of interacting mechanisms, with cellular senescence at its core. Targeted interventions addressing core hallmarks represent a critical direction in anti-aging research, offering theoretical and translational foundations for delaying aging and preventing age-related diseases.

However, the twelve hallmarks are largely descriptive and correlative rather than fully causal in humans. Many single-hallmark interventions succeed preclinically but fail clinically, revealing an oversimplified view of aging networks and a major translational bottleneck (López-Otín et al., 2023; Biga et al., 2025).

### 3.2 Representative anti-aging drugs and core mechanisms of action

In recent years, intervention strategies targeting cellular senescence have garnered widespread attention as therapeutic approaches to delay aging and extend healthspan. Current anti-aging therapies are broadly classified into two major categories: Senolytics (senescent cell clearance agents) and Senomorphics (senescent cell phenotype modulators) (Kirkland and Tchkonia, 2020). The former aims to selectively induce apoptosis in senescent cells, while the latter focuses on inhibiting the harmful components of their Senescence-Associated Secretory Phenotype (SASP) without eliminating the cells. Furthermore, emerging strategies in recent years also include Senoblockers (senescence blockers) and Senoreversers (senescence reversers) (Dhokia et al., 2024), as detailed in Table 1. These interventions aim to reduce senescent cell burden and modulate their secretory activity, thereby alleviating tissue dysfunction and enhancing organismal resilience to stress and disease. Current targeted strategies for representative anti-aging drugs are summarized in Table 1. Mechanistically, nearly all effective interventions converge on four evolutionarily conserved aging-related pathways: suppression of the SASP program, restoration of autophagy and mitophagy, inhibition of NF-κB/NLRP3 inflammasome signaling, and modulation of the mTOR/AMPK/SIRT1 metabolic axis, forming a unified regulatory network rather than isolated effects.

**TABLE 1:** Representative senotherapeutic drugs and their main strategies and mechanisms.

Drug category	Represent agents	Sources	Drug targets	Mechanism of action	Indications/Diseases	NCT number	Phase	Ref.
Senolytics	Dasatinib + Quercetin (D+Q)	Synthetic + Natural	Src family kinases/Eph receptors/PI3K/AKT	Inhibits pro-survival tyrosine kinases and PI3K/AKT pathway, induces senescence cell apoptosis	Idiopathic pulmonary fibrosis, Alzheimer's disease, diabetic kidney disease, chronic kidney disease, aging	NCT02652052, NCT02874989, NCT04063124, NCT04785300, NCT05422885	Phase I/II	Justice et al. (2019), Hickson et al. (2019)
Senolytics	Fisetin	Natural compound	p16/p21/PI3K/AKT/NF-κB	Modulates p16/CDK6 interaction and ROS pathway, induces senescent cell apoptosis	Knee osteoarthritis, femoroacetabular impingement syndrome, frailty syndrome, COVID-19	NCT04815902, NCT04770064, NCT05025956, NCT03675724, NCT04537299	Phase I/II	Yousefzadeh et al. (2018), Silva et al. (2024), Tavenier et al. (2024)
Senolytics	Navitoclax (ABT-263)	Chemical synthesis	BCL-2/BCL-xL/BCL-w	Blocks anti-apoptotic proteins, sensitizes senescent cells to apoptosis	Acute lymphoblastic leukemia, myelofibrosis, liver cirrhosis, small cell lung cancer	NCT03181126, NCT04468984, NCT02143401, NCT00445198	Phase I/II/III	Pemmaraju et al. (2022)
Senolytics	ABT-737	Chemical synthesis	BCL-xL/BCL-w	Blocks anti-apoptotic proteins, sensitizes senescent cells to apoptosis	Immune thrombocytopenia, age-related loss of liver regeneration	NCT00902018	Phase II / Preclinical	Yosef et al. (2016)
Senolytics	ABT-199 (Venetoclax)	Chemical synthesis	BCL-2	Selectively inhibits BCL-2, induces senescent cell apoptosis	Acute myeloid leukemia, kidney injury, breast tumor, hematopoietic stem cell transplantation	NCT01994837, NCT04810598, NCT03900884, NCT05583175	Phase I/II	Yosef et al. (2016)
Senolytics	UBX0101	Chemical synthesis	MDM2-p53/CDK4/6	Modulates cell cycle regulators and apoptotic checkpoints	Knee osteoarthritis	NCT04229225, NCT03513016	Phase I	Lane et al. (2021), Chin et al. (2023)
Senolytics	UBX1325	Chemical synthesis	MDM2-p53 pathway	Modulates cell cycle regulators and apoptotic checkpoints	Diabetic macular edema, neovascular age-related macular degeneration	NCT04537884	Phase I	(Khanani et al., 2025)
Senomorphics	Glucocorticoids (Dexamethasone)	Chemical synthesis	Glucocorticoid receptor/NF-κB	Inhibits SASP cytokine expression, broadly suppresses inflammation	Inflammatory diseases	Approved	Clinically approved / Repurposing debated	(Thrikawala et al., 2024)
Senomorphics	Rapamycin (Sirolimus)	Natural compound	mTORC1	Inhibits mTOR-mediated translation of SASP factors, improves immune function, reduces inflammation	Aging-related diseases	NCT02432287	Phase II	(Chrienova et al., 2022)
Senomorphics	Metformin	Natural compound	AMPK/NF-κB/mTOR	Activates AMPK, inhibits mTOR/NF-κB, indirectly suppresses SASP	Type 2 diabetes, aging	TAME trial NCT04245771	Phase II/III	(Barzilai et al., 2016; Pan et al., 2025)
Senomorphics	Ruxolitinib/Baricitinib	Chemical synthesis	JAK1/2	Blocks JAK-STAT signaling, inhibits IL-6/IL-8-mediated SASP amplification	Frailty, inflammation, myelofibrosis, rheumatoid arthritis	Not specified	Phase II	(Chen et al., 2021; Frede et al., 2023)
Senomorphics	Curcumin	Natural compound	NF-κB/Nrf2	Inhibits NF-κB pathway to reduce inflammatory cytokine release, downregulates Nrf2 pathway	Inflammation-related diseases	None	Preclinical / Dietary supplement	(Yang et al., 2024)
Senomorphics	Resveratrol	Natural compound	SIRT1/NF-κB	Activates SIRT1, inhibits NF-κB signaling and oxidative stress	Not specified	None	Dietary supplement / Limited clinical trials	(Hammad et al., 2021)
Senoblockers	Ibrutinib	Chemical synthesis	BTK	Interferes with p53 stability, blocks stress-induced senescence	Hematologic malignancies	Approved (for oncology)	Clinically approved (oncology)	(Das et al., 2025)
Senoreversers	Reversine	Chemical synthesis	Aurora B kinase	Inhibits Aurora B kinase, restores autophagy and mitochondrial function, improves metabolic features	Not specified	None	Preclinical	(Rajabian et al., 2023)
Immuno-related Senolytics	uPAR-directed CAR-T	Biological	uPAR	Engineered T cells recognize and clear uPAR-expressing senescent cells	Liver fibrosis, lung adenocarcinoma	None	Preclinical	(Amor et al., 2024)
Immuno-related Senolytics	NKG2D-CAR-T	Biological	NKG2D ligands	Engineered T cells recognize NKG2D ligands and clear senescent cells	Age-related pathologies	None	Preclinical (non-human primate)	(Yang et al., 2023)
Immuno-related Senolytics	GPNMB vaccine	Biological	GPNMB	Vaccination induces immune system to clear GPNMB-expressing senescent cells	Age-related phenotypes	None	Preclinical	(Mendelsohn and Larrick, 2022)
Energy metabolism modulator	Spermidine	Natural compound	Autophagy	Enhances autophagy, inhibits oxidative stress and necrosis	Cardiac and neuroprotection	None	Preclinical	(Satarker et al., 2024)
Energy metabolism modulator	Urolithin A	Natural compound	Mitophagy	Activates mitophagy, improves muscle function	Not specified	None	Preclinical	(Luan et al., 2021; Moussa et al., 2025)
NAD+ precursor/Sirtuin activator	NMN/NR	Natural/Synthetic	NAD+/Sirtuins	Raises NAD+ levels, activates sirtuins, improves mitochondrial function, enhances DNA repair	Not specified	None	Preclinical / Metabolic intervention studies	(Lin et al., 2025; Katayoshi et al., 2023)

Senolytics induce apoptosis in senescent cells by interfering with upregulated senescent cell anti-apoptotic pathways (SCAPs), thereby depriving them of survival dependence (Burdusel et al., 2024). Among representative compounds, Dasatinib + Quercetin (D + Q) is the first senolytic combination demonstrated to exhibit synergistic effects. It inhibits Ephrin-dependent tyrosine kinase signaling and the PI3K/AKT pathway, respectively, effectively clearing senescent preadipocytes and endothelial cells (Tang et al., 2025). Navitoclax, a BCL-2/BCL-xL inhibitor, shows robust senolytic activity but is limited by hematological toxicity including thrombocytopenia and neutropenia, restricting clinical application (Pemmaraju et al., 2022; Skov et al., 2021). Quercetin also exerts multi-target anti-aging effects by scavenging free radicals and enhancing cellular antioxidant capacity through activation of the Nrf2 pathway (Ouyang et al., 2016). The natural flavonoid Fisetin exerts senolytic effects by modulating p16/CDK6 interactions and ROS pathways. It effectively clears multiple types of senescent cells with relatively low toxicity, demonstrating promising clinical translation potential (Dong et al., 2025).

**FIGURE 3:**
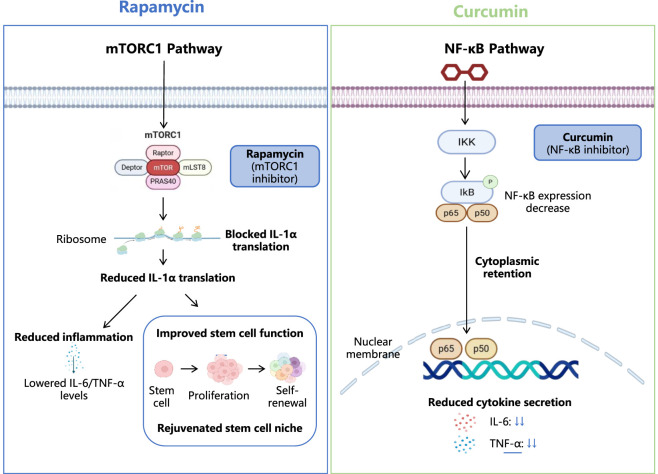
The inflammatory regulatory effects of rapamycin and curcumin in anti-aging.

Senomorphics (senescence phenotype modulators) mitigate SASP-mediated chronic inflammation and tissue remodeling effects by inhibiting core signaling pathways such as NF-κB, mTOR, and JAK/STAT (Saliev and Singh, 2025). Rapamycin, an mTORC1 inhibitor, effectively reduces inflammation levels and improves stem cell function by suppressing the translation of key SASP factors such as IL-1α (Pietrocola and Kroemer, 2017). However, long-term use carries risks of immunosuppression, hyperlipidemia, and insulin resistance, requiring careful risk–benefit balancing in clinical settings (Adav and Wang, 2021). Among natural products, curcumin demonstrates significant anti-inflammatory and anti-aging activities by inhibiting NF-κB pathway activity and reducing the release of inflammatory cytokines including IL-6 and TNF-α (Figure 3), but low bioavailability and poor reproducibility hinder consistent clinical efficacy (Ouyang et al., 2016). Resveratrol and its derivatives represent classical anti-aging compounds that primarily function through activation of the SIRT1 Pathway. However, their low oral bioavailability (approximately 1%) restricts clinical application. To address this limitation, synthetic derivatives such as acetylated resveratrol have been developed, demonstrating significantly enhanced stability and bioactivity (Nawaz et al., 2017).

Among emerging strategies, Senoblockers aim to prevent senescence initiation; for example, the BTK inhibitor Ibrutinib blocks stress-induced senescence by interfering with p53 stability, but *in vivo* evidence remains preliminary and clinical translation is distant (Dhokia et al., 2024; Das et al., 2025). Senoreversers aim to reverse established aging phenotypes. For instance, the small molecule Reversine improves the metabolic profile of senescent cells by restoring autophagy and mitochondrial function. Transient expression of certain transcription reprogramming factors (such as OSKM) can restore nucleocytoplasmic compartmentalization (NCC) in senescent cells, thereby reversing the senescent phenotype, however, potential tumorigenicity and limited controllability currently preclude clinical application (Rajabian et al., 2023; Dhokia et al., 2024). Furthermore, imbalances in metal ion metabolism are closely associated with aging. Studies reveal that selenium content in the lenses of elderly individuals significantly increases with age (2.3 times higher in individuals over 60 compared to those under 20), while copper content decreases (by 38%). These alterations may promote cataract formation by inducing oxidative stress, indicating that maintaining metal ion homeostasis represents a potential target for anti-aging interventions (Langford-Smith et al., 2016).

Overall, interventions supported by robust, reproducible evidence include rapamycin, metformin, D + Q, fisetin, and navitoclax. In contrast, senoblockers, senoreversers, and most reprogramming-based strategies remain speculative and require further mechanistic validation and stringent safety evaluation.

### 3.3 Current research status in epidemiology of anti-aging drugs

Epidemiological studies on anti-aging drugs primarily focus on population usage patterns, clinical efficacy evaluation, and safety assessment, providing crucial data support for their clinical translation. Among elderly populations, dietary supplements are the most common anti-aging interventions. For example, nucleotide supplements were used in the TALENTs Trial to assess their impact on physiological indicators in older adults, which enrolled 122 participants aged 60–70 years, with 67.21% being female, and the majority having at least one chronic disease (Wang et al., 2024). In terms of drug usage, epidemiological studies on Aspirin as a potential anti-aging drug demonstrate that long-term low-dose administration reduces cardiovascular disease risk (RR = 0.79, 95% CI 0.71–0.88) but increases bleeding risk (OR = 1.54, 95% CI 1.32–1.80). This indicates that clinical application requires strict dosage control and personalized risk assessment (Lushchak et al., 2021).

Traditional Chinese Medicine has gained attention in anti-aging applications due to its multi-target effects and low toxicity profile. For instance, Polygonum multiflorum extract (PME) significantly reduced IL-4 and IL-5 cytokine levels (by 45% and 52%, respectively) through Th2 cell activation inhibition in asthmatic mouse models, while simultaneously improving airway inflammation. These effects indirectly demonstrate its anti-aging-related anti-inflammatory potential (Lee C. C. et al., 2017). In the field of skin anti-aging, asiaticoside significantly improves wrinkles and elasticity in photoaged skin (30% reduction in wrinkle depth, 25% increase in elasticity) by promoting collagen synthesis and inhibiting Matrix Metalloproteinases (MMP) activity, demonstrating promising prospects for clinical application (Sikdar et al., 2016). Notably, epidemiological applications of anti-aging drugs exhibit regional variations. For instance, in Asian countries, the utilization rate of Traditional Chinese Medicine is significantly higher than in Western nations, whereas in Europe and America, basic research and clinical trials on synthetic compounds (such as the aforementioned Rapamycin) are more concentrated (Fang et al., 2020).

### 3.4 Application framework of multi-omics technologies in anti-aging Drug research

Building upon the aforementioned mechanisms and epidemiology, multi-omics-based drug screening technology integrates multi-dimensional molecular data including genomics, transcriptomics, and metabolomics. This integration enables comprehensive analysis of drug mechanisms of action and provides core technical support for the efficient screening of anti-aging drugs.

In natural product research, multi-omics analysis of Tiger Milk Mushroom reveals that its constituents—including polysaccharides and terpenoids—exert multi-target anti-aging effects by modulating oxidative stress, inflammatory responses, and gut microbiota homeostasis (Wong et al., 2024). Multi-omics integrated analysis (UHPLC-Q-TOFMS and RNA-seq) of Lycium ruthenicum anthocyanins (LRA) demonstrated that redirecting carbon metabolism towards polysaccharide precursor synthesis (e.g., UDP-glucose, UDP-galactose) through TCA cycle inhibition, while promoting antioxidant production, enhances cell membrane fluidity and toxic substance degradation capacity, ultimately improving mycelial cell anti-aging activity (Jiaofen et al., 2024). Multi-omics analysis further revealed that specific oligosaccharides significantly reduced pro-inflammatory cytokine levels in aging mouse serum (TNF-α by 35%, IL-6 by 42%) (Chen et al., 2024).

In clinical research, multi-omics technologies are extensively employed to evaluate the efficacy of anti-aging interventions. The TALENTs Trial (Targeting Aging and Longevity with Exogenous Nucleotides) administered nucleotide interventions to elderly participants aged 60–70, collecting blood samples for multi-omics sequencing to analyze effects on telomere length, DNA methylation age, and immune-inflammatory markers (Wang et al., 2024). Furthermore, multi-omics integration strategies demonstrate substantial advantages in biological age calculation. Common integration approaches include concatenated machine learning (modeling after merging multi-omics data matrices), fusion models (integrating multiple omics-based submodels), and AI transformation methods (modeling omics data converted into graph structures). These methods significantly improve biological age prediction accuracy, providing more precise quantitative metrics for anti-aging efficacy assessment (Solovev, 2024). In skin aging research, multi-omics technologies have revealed molecular differences between intrinsic aging (epidermal thinning and reduced elasticity) and extrinsic aging (deepened wrinkles and pigmentation). Transcriptomics analysis indicates that epidermal differentiation genes (e.g., KRT10) are downregulated in intrinsic aging, while matrix metalloproteinases (e.g., MMP1) are upregulated in extrinsic aging (Long et al., 2025).

In addition to elucidating molecular changes associated with aging, a principal application of multi-omic strategies resides in the identification of aging biomarkers. Through the integrative analysis of genomic, transcriptomic, proteomic, metabolomic, and microbiomic data, multi-omic pipelines enable the capture of molecular fingerprints that reflect an individual’s biological age trajectory, predisposition to disease, and pharmacological responsiveness. Such biomarkers not only support aging subtyping and longitudinal surveillance of treatment effects but also furnish essential baseline datasets for subsequent *in silico* modeling and the rational design of precision interventions. Despite advances, multi-omics approaches suffer from poor standardization, high heterogeneity, limiting real-world utility (Skov et al., 2021; Odenkirk et al., 2021).

### 3.5 Advances in multi-omics-based anti-aging biomarker research

Multi-omics technologies, leveraging their high-throughput and systematic characteristics, provide powerful tools for efficient screening and precise identification of aging biomarkers, serving as core methods for assessing aging progression and predicting age-related disease risks. These biomarkers enable aging phenotype stratification, disease risk prediction, intervention efficacy evaluation, and personalized anti-aging therapy (Table 2).

**TABLE 2:** Key multi-omics biomarkers of aging: From mechanisms to clinical application.

Biomarker category	Omics platform	Representative biomarkers	Associated aging mechanisms	Potential clinical application	Major limitations	Key references
Epigenetic Clocks	Methylomics	Horvath clock, Hannum clock, PhenoAge, GrimAge, DunedinPACE	Epigenetic alterations; Cellular senescence; Integrative aging signals	Biological age prediction; All-cause mortality risk assessment (GrimAge HR≈1.56); Monitoring anti-aging interventions	Population bias (trained primarily on European cohorts); Causality unclear; Reduced accuracy across ethnic groups	Yamada, 2025; Wu et al., 2025; Horvath, 2013
Proteomic Aging Signatures	Proteomics	11-protein signature (GDF15, MMP12, TNFRSF1A, etc.); SOMAscan/Olink platforms	Altered intercellular communication; Chronic inflammation; Mitochondrial dysfunction	Biological age estimation; Identification of individual “ageotypes”; Frailty risk assessment	High cost; Significant batch effects; Need for cross-cohort external validation	Adav and Wang, 2021
SASP Factors	Transcriptomics/Proteomics	IL-6, IL-8, IL-1β, MMPs (MMP1, MMP3, MMP9), PAI-1, CXCL1	Cellular senescence; Chronic inflammation (inflammaging); Altered intercellular communication	Monitoring senolytic efficacy (e.g., D+Q, Navitoclax); Disease progression assessment (e.g., OA, IPF)	Not exclusive to senescent cells; Influenced by acute inflammation; Lack of standardized cut-off values	Kirkland and Tchkonia, 2020; López-Otín et al., 2023
Inflammaging Markers	Proteomics/Clinical biochemistry	hs-CRP, IL-6, TNF-α, Fibrinogen	Chronic inflammation; Immune dysregulation; Dysbiosis	Assessment of systemic low-grade inflammation; Cardiovascular aging risk prediction; Monitoring anti-inflammatory interventions (e.g., aspirin)	Low specificity; Confounded by obesity, infection, autoimmune diseases	Gaylord et al., 2023; Lushchak et al., 2021
Metabolomic Frailty Signatures	Metabolomics	Branched-chain amino acids (BCAA); Kynurenine; Tryptophan; Phosphocholine; AMP/ATP ratio	Deregulated nutrient-sensing; Mitochondrial dysfunction; Stem cell exhaustion	Frailty index assessment; Sarcopenia risk prediction; Guidance for personalized dietary/nutritional interventions	Dynamic fluctuations; Strongly influenced by sampling time and dietary status; Incomplete metabolite databases	Phan et al., 2023; Adav and Wang, 2021
Microbiome-Derived Aging Markers	Metagenomics/16S rRNA	Microbial diversity (Shannon index); *Akkermansia muciniphila* abundance; *Bifidobacterium* abundance; SCFA synthesis pathways	Dysbiosis; Chronic inflammation; Deregulated nutrient-sensing	Assessment of gut aging status; Guidance for probiotic/prebiotic interventions; Prediction of inflammaging risk	High geographic and dietary variability; Unclear causality; Insufficient functional gene analysis	Liu F. et al., 2025; López-Otín et al., 2023
Aging-Related Non-Coding RNAs	Transcriptomics	lncRNAs (MALAT1, NEAT1); circRNAs; miRNAs (miR-34a, miR-146a)	Epigenetic alterations; Altered intercellular communication; Mitochondrial dysfunction	Novel circulating biomarkers; Early aging warning; Potential therapeutic targets	Insufficient functional validation; Lack of detection standardization; Stability concerns	Han et al., 2025; You et al., 2016

Epigenetic clocks (e.g., Horvath clock, GrimAge) predict biological age by analyzing genome-wide DNA methylation patterns, demonstrating significantly higher accuracy than chronological age. Notably, GrimAge shows a stronger association with all-cause mortality (HR = 1.56) compared to chronological age (HR = 1.21), suggesting its potential as a prognostic biomarker for assessing aging-related mortality risk (Yamada, 2025). Metabolomics studies reveal that serum metabolites such as phosphocholine and the AMP/ATP ratio increase significantly with age, potentially serving as biomarkers of aging, which reflect mitochondrial dysfunction, oxidative stress, and metabolic imbalance. (Phan et al., 2023). Transcriptomics-level analysis of aging-related gene expression profiles demonstrates upregulated expression of inflammation-associated genes (e.g., IL-6, TNF-α), inducing chronic low-grade inflammation (inflammaging), while downregulated expression of cellular repair genes (e.g., SIRT1, FOXO3) accelerates aging progression. Proteomics research reveals that alterations in serum protein profiles constitute important biomarkers of aging. Serum C-reactive protein (CRP) and fibrinogen levels increase with advancing age, whereas albumin and apolipoprotein A1 concentrations decline (Adav and Wang, 2021). Furthermore, compositional changes in gut microbiota are closely associated with aging. In elderly populations, Akkermansia abundance significantly decreases while *Enterococcus* abundance abnormally increases, demonstrating that such microbial imbalances affect host aging through metabolites (Liu F. et al., 2025).

In non-human primate models, multi-omics integrated analysis revealed that lncRNAs exhibit high expression during critical aging periods, with their expression profiles closely linked to cancer-related signaling pathways. Concurrently, aging processes are regulated by serum exosomal proteins through metabolic pathways (Liu et al., 2024).

Despite rapid progress in multi-omics biomarker discovery, several critical challenges remain to be addressed (Tiwari and Pandey, 2023). First, multi-omics data suffer from high heterogeneity and prominent batch effects caused by platform differences, preanalytical variation, and cohort diversity, which severely compromise cross-study reproducibility. Second, the lack of standardized protocols for sample collection, processing, normalization, and statistical modeling hinders clinical translation and large-scale application. Third, most current biomarkers represent associations rather than causal relationships with aging, limiting their mechanistic value and target validation. Fourth, population bias exists in many widely used signatures, which are primarily trained on European cohorts and show reduced accuracy in other ethnic groups. Finally, the clinical translation gap remains substantial, as few biomarkers have been validated in large prospective randomized controlled trials as surrogate endpoints for anti-aging drug development and clinical approval. Future research should prioritize multicenter long-term cohort follow-up, standardized protocols for sample collection and data harmonization, as well as cross-population validation, in order to enhance the clinical applicability of aging biomarkers.

Beyond these technical challenges, a more fundamental challenge lies in the uncertainty surrounding the concept of “biological age” itself (López-Otín et al., 2023; Yamada, 2025). Different aging clocks—including epigenetic clocks (e.g., Horvath, GrimAge), transcriptomic clocks, and proteomic clocks—often yield inconsistent estimates of biological age when applied to the same individual or population (Long et al., 2025). For example, an individual may show accelerated aging according to an epigenetic clock but appear younger by a proteomic or transcriptomic clock. These discrepancies arise from differences in the underlying molecular layers captured (DNA methylation vs. gene expression vs. protein abundance), the training cohorts used, the algorithmic approaches employed, and the biological processes each clock prioritizes (e.g., inflammation, cellular senescence, metabolic dysregulation) (Solovev, 2024). Consequently, there is currently no universally accepted framework for defining or measuring biological aging (Wu et al., 2025). This lack of consensus complicates the validation of anti-aging interventions, as the choice of clock can dramatically influence the apparent efficacy of a given drug (Patel et al., 2020). Future efforts must focus on cross-clock standardization, multi-omics integration, and prospective validation against hard clinical endpoints (e.g., morbidity, mortality) to establish a robust and clinically meaningful definition of biological age (Solovev, 2024; Yamada, 2025).

### 3.6 The technical role of artificial intelligence in anti-aging Drug discovery

AI relies entirely on multi-omics data to decode aging biology, identify aging-specific signatures, and prioritize therapeutic targets. Artificial Intelligence Technology plays a crucial role in Anti-Aging Drug Screening, particularly in core areas such as Target Discovery and Compound Optimization. The BioGPT model based on large language models (LLMs), trained on extensive biomedical literature data, successfully predicted potential anti-aging targets like CCR5 and PTH that had not been explicitly associated with the aging process, providing innovative approaches for novel target discovery (Zagirova et al., 2023). Another study employed the AI-driven PandaOmics platform to analyze transcriptomic data from numerous healthy and cancer samples, revealing shared key signaling pathways between aging and cancer, and identified 51 cancer targets with anti-aging potential, among which KDM1A was experimentally validated to significantly extend the lifespan of *Caenorhabditis elegans* (Pun et al., 2023).

In drug discovery, AI technology accelerates the development and molecular structural optimization of natural products. Ginkgetin was predicted to target the STING protein via graph convolutional networks, with subsequent experiments confirming its direct binding to STING’s carboxy-terminal domain. This interaction inhibits STING activation and downstream signaling, thereby alleviating systemic inflammation in aging mice and demonstrating significant anti-aging potential (Liu Y. et al., 2025). Moreover, AI has made substantial breakthroughs in anti-aging peptide prediction, with the Antiaging-FL model achieving an AUC value of 1.00 on the AAP400 *in vitro* dataset and significantly enhancing screening efficiency for anti-aging peptides (Zhang et al., 2025). In model organism research, the convolutional neural network-based WormNet achieves over 85% accuracy in predicting lifespan classifications through analysis of *Caenorhabditis elegans* images, providing an efficient tool for rapid screening of anti-aging drugs (Galimov and Yakimovich, 2022).

The reliability and generalizability of artificial intelligence-driven aging prediction models are fundamentally predicated on the robustness and replicability of the underlying biomarker datasets. Therefore, the integration of longitudinally validated multi-omic signatures with interpretable artificial intelligence frameworks constitutes a core direction for future translational aging research. However, AI models are limited by population bias, low interpretability, and poor generalization. Most predictions lack experimental validation, and “black box” structures impede mechanistic confidence and regulatory acceptance (Pham et al., 2025; Wu et al., 2025).

## 4 Screening and evaluation methods for anti-aging drugs

### 4.1 Multi-omics-based screening technologies for anti-aging drugs

Multi-omics profiling constructs the fundamental data landscape for AI-powered aging signature decoding and anti-aging drug mining by integrating multi-layered molecular evidence across genomic, transcriptomic, proteomic, and metabolomic dimensions to systematically decipher drug action mechanisms and provide core technical support for high-efficiency anti-aging drug screening. By integrating multi-layered molecular evidence across genomic, transcriptomic, proteomic, and metabolomic dimensions, multi-omics technologies systematically decipher drug action mechanisms and provide core technical support for high-efficiency anti-aging drug screening. In cellular models, multi-omics technologies are widely employed to screen for anti-aging compounds. For instance, analysis of transcriptome and metabolome alterations in senescent cells revealed that resveratrol reverses aging-associated gene expression profiles (e.g., up-regulating SIRT1 and down-regulating p21), thereby exerting anti-aging effects (You et al., 2016).

In preclinical studies, multi-omics technologies have accelerated drug target discovery and validation. Analysis of transcriptome and metabolome data from liver and adipose tissues in aging mice demonstrated that a combination of vitamin D and resveratrol significantly improved insulin resistance (achieving a 28% reduction in blood glucose and a 35% decrease in insulin levels) (Rui et al., 2017). In drug screening platforms, ViaChip based on Microfluidic Technology separates live cells from senescent cells through cell size differences, achieving an enrichment efficiency of 67%, thereby providing a novel tool for high-throughput screening of anti-aging drugs (Yeh et al., 2022). Furthermore, multi-omics integration strategies demonstrate significant advantages in drug repositioning. Analysis of aging-related gene expression profiles revealed that metformin exerts anti-aging effects by activating the AMPK pathway, providing a scientific basis for the clinical repositioning of metformin (Pietrocola and Kroemer, 2017).

### 4.2 Artificial intelligence-assisted Drug discovery and molecular structural optimization

AI models integrate multi-omics-derived aging signatures to improve target prediction, virtual screening, and molecular design. Building on earlier AI-based target discovery (e.g., targets such as CCR5, PTH, and KDM1A identified by platforms like BioGPT and PandaOmics) (Zagirova et al., 2023; Pun et al., 2023), AI Technology now plays a pivotal role in drug optimization and Virtual Screening, significantly reducing the R&D cycle and R&D Costs.

In Virtual Screening, the DrugPipe platform enables Drug Repositioning for unknown targets by integrating generative models and binding pocket prediction. Its performance is comparable to that of the traditional screening tool QVina-W, but with significantly reduced computational time, markedly improving Virtual Screening efficiency (Pham et al., 2025). Additionally, AI technology has been employed for molecular structural optimization of drug compounds. Through the use of generative adversarial networks (GAN), it designs resveratrol derivatives with enhanced stability and bioactivity, effectively improving their oral bioavailability and resolving the critical bottleneck for resveratrol’s clinical application, with no clinical validation yet (You et al., 2016). In natural product screening, AI algorithms can rapidly identify compounds with potential anti-aging activity by analyzing structural characteristics and activity data of natural products. For example, Smer-Barreto et al. utilized an XGBoost machine learning model to screen thousands of compounds, identifying natural products such as Ginkgo biloba biflavones and Periplocin, and subsequently validated their significant anti-aging activity (Smer-Barreto et al., 2023).

Therefore, Emerging computational models have further advanced the integration of multi-omics data, demonstrating how machine learning frameworks can enhance biological age prediction and drug target prioritization (Tiwari, B. and Pandey, K. (2023). Future research should prioritize multicenter longitudinal cohort follow-up, standardized protocols for sample collection and data harmonization, as well as cross-population validation, to enhance the clinical applicability of aging biomarkers. Furthermore, adherence to FAIR data principles and the adoption of federated learning frameworks across international consortia will be critical for overcoming data heterogeneity and improving the generalizability of multi-omics-derived biomarkers (Wilkinson et al., 2016; Rieke et al., 2020).

### 4.3 Preclinical evaluation system for anti-aging drugs

The preclinical evaluation of anti-aging drugs is a critical stage in drug development. Its core objectives are to verify the anti-aging activity, mechanism of action, and safety profile of the drug, providing a scientific foundation for clinical trials. Current preclinical evaluation methods primarily include *in vitro* cell models, model organism models, and organ-on-a-chip models, collectively forming a multi-dimensional and multi-tiered evaluation system.

In vitro models, SH-SY5Y neuroblastoma cells are used to evaluate the neuroprotective effects of compounds, assessing their impact on neuronal apoptosis, ROS production, and the expression of related genes (Ramkumar et al., 2017). In model organisms, *Caenorhabditis elegans* and *Drosophila melanogaster* are commonly employed screening models for anti-aging drugs. For example, KDM1A knockdown extends *Caenorhabditis elegans* lifespan, while Pleurotus tuber-regium extracts prolong *Drosophila melanogaster* lifespan and enhance motor function (Wong et al., 2024; Pun et al., 2023). In organ-on-a-chip models, microfluidic technology platforms simulate the microenvironment of aging tissues, overcoming limitations of traditional cellular and animal models. Retinal chip models evaluate anti-aging drug protection effects on retinal pigment epithelial cells, providing more physiologically relevant environments for drug screening (Rodboon et al., 2022).

Pharmacokinetics and toxicology studies constitute central components in preclinical safety evaluations. Pharmacokinetic studies of FTY720-C2 and FTY720-Mitoxy demonstrate favorable blood-brain barrier penetration, offering advantages for anti-aging therapies targeting neurodegenerative diseases (Enoru et al., 2016). The Geropathology Grading Platform (GGP) evaluates drug efficacy in ameliorating aging-associated pathologies through standardized histopathological scoring, revealing that rapamycin significantly reduces liver fibrosis scores in aged mice (Ladiges et al., 2017).

Whereas, A critical limitation is the weak concordance between preclinical models and human aging. Most systems fail to recapitulate human pathophysiology, leading to low translational predictivity (Rodboon et al., 2022; Rui et al., 2017).

## 5 Clinical translation and therapeutic strategies of anti-aging drugs

### 5.1 Clinical application and efficacy of existing anti-aging drugs

Existing anti-aging drugs primarily include traditional Chinese medicine, natural products, and synthetic compounds. Their clinical applications encompass multiple fields such as systemic anti-aging, organ-specific anti-aging, and treatment of age-related diseases, with variations in efficacy and safety. Among traditional Chinese medicines, ginseng extracts activate the AMPK pathway to ameliorate aging-related metabolic disorders and significantly improve insulin sensitivity, demonstrating favorable anti-aging effects in the elderly population (Lee Y. et al., 2017). Rapamycin, as a classic mTOR inhibitor, extends lifespan by 20%–30% in animal models; however, its clinical application is limited by immunosuppressive side effects, restricting its use primarily to specific populations for anti-aging interventions (Adav and Wang, 2021). Although Resveratrol activates the SIRT1 Pathway, its clinical efficacy is contentious. A clinical trial indicated it reduces glycated hemoglobin by 0.5% but fails to significantly improve insulin resistance, suggesting its clinical application requires further optimization of dosing regimens (Tomaciello et al., 2016).

In skin anti-aging applications, Vitamin C increases collagen by 25% and reduces wrinkle depth by 30% in small-scale clinical cohorts (Foster et al., 2024). In neurodegenerative diseases, memantine improves cognitive function in Alzheimer’s patients by modulating NMDA receptors, increasing MMSE scores by 2.5 points, and provides an important therapeutic option for aging-related neurodegenerative diseases (Alonso Abreu et al., 2018). Additionally, cell therapies such as mesenchymal stem cell transplantation demonstrate significant anti-aging potential. Exosomes derived from human umbilical cord MSCs improve cognitive function in aged mice and reduce neuroinflammation, offering a novel technical approach for anti-aging interventions (Zhang et al., 2024).

### 5.2 Personalized anti-aging therapy guided by multi-omics

Multi-omics technologies provide a scientific foundation for personalized anti-aging therapy. By analyzing multi-dimensional data such as an individual’s genome, transcriptome, and metabolome, specific aging characteristics and potential risks can be identified, enabling precise interventions. The epigenetic clock can predict an individual’s biological age with significantly higher accuracy than chronological age, providing a core basis for developing personalized anti-aging intervention strategies (Yamada, 2025). Metabolomics biomarkers serve as potential indicators of aging and guide personalized dietary interventions and drug selection (Phan et al., 2023). Gut microbiota analysis identifies individual microbial composition characteristics, enabling development of targeted probiotic intervention plans. Supplementation with Akkermansia improves intestinal barrier function in aged mice and delays the aging process (Liu F. et al., 2025).

In clinical practice, Multi-omics-guided Personalized Treatment has achieved initial progress. For patients with Age-related Macular Degeneration, genomic data analysis reveals that CFH gene variants correlate with disease risk, allowing targeted use of Complement Inhibitors to enhance therapeutic efficacy (Biga et al., 2025). In diabetic patients, transcriptomic data analysis demonstrates that SIRT1 expression levels correlate with Insulin Resistance, facilitating development of personalized intervention plans involving Resveratrol and other agents based on SIRT1 expression profiles (Phan et al., 2023). The TALENTs Trial also fully demonstrates the application value of Multi-omics in evaluating the impact of Nucleotide Supplements on elderly populations (Wang et al., 2024).

Personalized anti-aging strategies remain premature. Most biomarkers are not clinically validated or standardized. Biological age clocks are limited by population bias, and high costs and regulatory barriers hinder clinical adoption (Yamada, 2025; Patel et al., 2020).

### 5.3 Application of artificial intelligence in clinical trials of anti-aging drugs

AI Technology is primarily employed in clinical trials of Anti-aging Drugs for patient recruitment, efficacy prediction, and safety monitoring, significantly enhancing trial efficiency, accuracy, and safety while facilitating clinical translation. For patient recruitment, AI Algorithms rapidly identify eligible participants by analyzing electronic health records. In the TALENTs Trial, an AI Algorithm screened 122 eligible elderly subjects from 301 potential candidates, substantially shortening recruitment cycles and reducing costs (Wang et al., 2024). In efficacy prediction, AI models analyze multi-omics data to predict patient drug responses, achieving 85% accuracy (Pun et al., 2023).

For safety monitoring, AI algorithms analyze clinical trial data in real-time to rapidly identify potential adverse reactions; for instance, natural language processing analysis of adverse event reports revealed that Aspirin’s bleeding risk correlates closely with dosage, providing reference for clinical dose adjustment (Dong et al., 2025). Furthermore, AI technology optimizes clinical trial design by simulating different dosages and administration regimens, reducing trial sample size and duration while lowering R&D costs (Jin and Jin, 2025). In clinical trials for skin anti-aging, AI algorithms assessed drug efficacy by analyzing skin images, achieving an accuracy of 90% (Kennedy et al., 2020). AI in clinical trials is limited by small cohorts, overfitting, and poor external validation. Regulatory acceptance of AI-derived endpoints and patient stratification remains limited (Jin and Jin, 2025; Kennedy et al., 2020).

## 6 Future perspectives of anti-aging Drug research leveraging multi-omics and artificial intelligence

### 6.1 Deep integration of multi-omics and artificial intelligence

The deep integration of multi-omics and AI represents the foundational paradigm unifying aging biology and drug discovery, which will bring revolutionary changes to anti-aging drug research. In target discovery, AI algorithms can integrate multi-omics data to overcome the limitations of traditional data integration methods, thereby identifying novel anti-aging targets. For example, analysis based on the PandaOmics platform revealed KDM1A as a potential anti-aging target (Pun et al., 2023). Future research will develop more efficient multi-omics data integration methods. For example, Graph Neural Network-based models can simultaneously process omics data of different types and dimensions, overcoming the limitations of traditional approaches in handling data heterogeneity and improving target accuracy (Shao et al., 2024). In drug screening, AI algorithms can rapidly identify potential anti-aging compounds through virtual screening. For instance, the DrugPipe platform enables drug repositioning for unknown targets, while generative AI can design novel-structure anti-aging molecules, significantly enhancing the efficiency and innovation of drug screening (Pham et al., 2025). For clinical trials, AI algorithms can optimize trial design while improving efficiency and accuracy through real-time data monitoring (Jin and Jin, 2025).

In the future, the integration of multi-omics and artificial intelligence will be underpinned by standardized methodological frameworks to overcome data heterogeneity. These include harmonized experimental and computational pipelines, privacy-preserving federated learning for multicenter data integration, and FAIR-compliant data management practices (Rieke et al., 2020; Xu et al., 2021; Sansone et al., 2019). Collectively, these strategies will improve the reliability of cross-platform, cross-cohort, and cross-population data integration, facilitating the identification of generalizable aging biomarkers and AI-driven therapeutic targets. Ultimately, they will accelerate the translation of multi-omics discoveries into clinically effective anti-aging interventions.

### 6.2 Application of emerging technologies and novel Drug targets in anti-aging drugs

Regarding novel drug targets, future anti-aging strategies increasingly rely on the integration of multi-omics, AI, and aging biology to prioritize mechanistically convergent and evolutionarily conserved targets. These key targets mainly include aging-associated noncoding RNAs (lncRNAs, circRNAs), epigenetic regulators, and gut–immune axis components, which collectively govern cellular senescence, chronic inflammation, and metabolic deterioration (Han et al., 2025). Furthermore, emerging technologies including gene editing, cell therapy, and nanotechnology will open new avenues for anti-aging drug research, propelling anti-aging therapeutics into a new phase.

In the field of gene editing, CRISPR-Cas9 technology can repair aging-related genetic mutations. For example, editing the SIRT1 gene extends the lifespan of mice, providing precise genetic interventions for anti-aging therapy, but off-target risks, limited delivery efficiency, and ethical constraints hinder clinical translation (Zhang et al., 2024). In cell therapy, approaches such as MSCs transplantation have demonstrated anti-aging potential in preclinical studies (Lee Y. et al., 2017). In nanotechnology, nanocarriers enhance the bioavailability and targeting of anti-aging drugs. For instance, Resveratrol nanoformulations increase oral bioavailability to over 10%, addressing the challenge of low bioavailability in traditional drugs (You et al., 2016).

Furthermore, Organ-on-a-chip and organoid models recapitulate human tissue microenvironments more faithfully than traditional animal models, improving the predictive validity of preclinical screening and reducing species mismatch (Rodboon et al., 2022; Rui et al., 2017). Synthetic biology-driven cellular reprogramming reverses senescence by resetting epigenetic and metabolic states; however, long-term safety and precise controllability require extensive validation before clinical implementation (Lee and Lee, 2022).

Clinically, the most strongly supported interventions include mTOR inhibition, senolytics (D + Q, UBX1325), AMPK activation (metformin), and anti-inflammatory approaches (aspirin, BTK inhibitors) (Lushchak et al., 2021; Pietrocola and Kroemer, 2017; Khanani et al., 2025). In contrast, most AI-generated molecules, epigenetic reprogramming strategies, and novel natural products remain mechanistically promising but clinically unproven.

In summary, the most clinically relevant and mechanistically robust anti-aging interventions converge on restoring autophagy/mitophagy, suppressing inflammaging, improving mitochondrial fitness, and reversing stem cell exhaustion. Future breakthroughs will depend on targeting these conserved pathways, improving reproducibility, mitigating side effects, and establishing rigorously validated clinical biomarkers of biological age (Alonso Abreu et al., 2018).

### 6.3 Popularization of personalized anti-aging therapy

**FIGURE 4:**
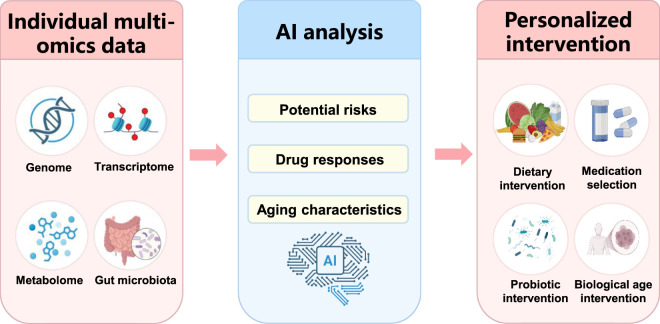
Multi omics guidance strategy for personalized anti-aging therapy.

With the decline in multi-omics testing costs and the refinement of AI models, personalized anti-aging therapy is expected to achieve widespread clinical adoption. Analysis of multi-omics data—including an individual’s genome, transcriptome, metabolome, and gut microbiota—enables precise assessment of aging characteristics, potential risks, and drug responses, thereby facilitating targeted intervention strategies (Nawaz et al., 2017; Sikdar et al., 2016). The epigenetic clock can accurately predict biological age, providing a fundamental basis for developing intervention plans (Nawaz et al., 2017), while metabolomics biomarkers guide personalized dietary interventions and drug selection. Gut microbiota analysis enables the formulation of targeted probiotic intervention strategies, thereby achieving multidimensional and personalized anti-aging interventions (Langford-Smith et al., 2016; Sikdar et al., 2016) (Figure 4). In the future, with declining costs of multi-omics testing and continuous refinement of AI models, personalized anti-aging therapy is expected to transition from research to routine clinical practice.

### 6.4 Policy regulatory frameworks and interdisciplinary collaboration mechanisms

The healthy advancement of anti-aging drug research necessitates robust policy regulation and efficient interdisciplinary collaboration. In policy regulation, future efforts will strengthen regulatory frameworks by establishing dedicated oversight systems for anti-aging drugs, standardizing their development, production, and marketing. For instance, the US FDA has initiated evaluations of regulatory frameworks for anti-aging drugs, defining efficacy and safety assessment criteria (Wu et al., 2025). Simultaneously, enhancing international cooperation through platforms such as the International Research Consortium on Aging and Disease (IRCAD) will facilitate multinational collaborative research, enabling data and technology sharing to jointly advance the development and clinical translation of anti-aging drugs (Fang et al., 2020). Furthermore, increased investment in multi-omics and AI technology R&D will be prioritized through policies such as tax incentives and research subsidies to encourage enterprises and research institutions to conduct relevant studies; Concurrently, public education will be enhanced to improve scientific understanding of aging and anti-aging drugs, guide rational consumption choices, and eliminate false advertising (Fang et al., 2020; Wong et al., 2024; Hofmann, 2023).

## 7 Ethics and challenges in anti-aging Drug research

### 7.1 Ethical issues and social impact of anti-aging drugs

Anti-aging drug research faces multiple ethical challenges including fairness, safety, and social implications, which constitute significant constraints on its healthy development. Regarding fairness, the high cost of drugs (such as Rapamycin) may exacerbate social inequality, leading to only high-income individuals accessing anti-aging treatments and raising ‘health inequality’ concerns (Hofmann, 2023). The expensive multi-omics sequencing and AI-assisted personalized intervention schemes further widen the longevity gap between high-income and disadvantaged populations; without tiered pricing and medical insurance coverage policies, equitable access to geroprotective therapies cannot be guaranteed for all demographic groups.

In terms of safety, long-term use of anti-aging drugs may pose unknown side effects; as outlined in section 1.1.3, both Aspirin and Rapamycin present documented safety risks with prolonged use (Justice et al., 2019). Most current clinical trials only implement short-term follow-up, lacking decades-long real-world safety data to capture cumulative toxicities such as immune suppression and oncogenic risks induced by chronic administration, which highlights an urgent demand for standardized long-term post-market monitoring systems and stratified surveillance for vulnerable groups like the elderly and immunocompromised patients.

Additionally, whether ‘Life Extension’ aligns with natural laws and whether it might intensify the social burden from population aging has sparked widespread ethical controversy (Gaille et al., 2020). The large-scale popularization of life-extending therapies will further increase the proportion of elderly populations, aggravate pension and medical care burdens, and trigger disputes over the definition of “normal physiological aging”, which may stigmatize groups unable to afford anti-aging interventions.

In terms of social impact, anti-aging drugs may reshape the labor market and retirement systems while introducing new challenges such as ‘age discrimination’, exerting profound effects on societal structures (Gaille et al., 2020; Hofmann, 2023). New forms of age stratification driven by differential access to longevity treatments will also force society to redefine retirement age, labor rights and age-related social welfare mechanisms.

Currently, the absence of specific regulations for anti-aging drugs has resulted in a proliferation of unverified products in the market, with prominent issues including false advertising and exaggerated efficacy claims, severely impeding the industry’s healthy development (Wu et al., 2025; Bajenova et al., 2019).

### 7.2 Limitations of multi-omics and artificial intelligence technologies

The limitations of multi-omics technologies primarily encompass data integration challenges, insufficient standardization, and high costs. Regarding data integration, the heterogeneity among different omics datasets presents significant integration challenges, impeding the efficient fusion and in-depth analysis of multi-omics data. Correlating DNA Methylation data with Transcriptomic data, for instance, necessitates complex bioinformatic methods (Odenkirk et al., 2021).

Beyond integration, batch effects from sequencing platforms and pre-analytical variables persist even after correction algorithms, leading to spurious aging signatures (e.g., “inflammaging” reflecting seasonal variation) (Gaylord et al., 2023). Cohort heterogeneity and population bias further limit generalizability. Epigenetic clocks trained on European-ancestry cohorts (UK Biobank, GTEx) show 30%–50% increased error in non-European populations (Wu et al., 2025), challenging universal anti-aging drug development. The scarcity of longitudinal data remains a critical bottleneck. Most studies are cross-sectional, unable to distinguish true aging trajectories from inter-individual differences, which constrains causal inference and surrogate endpoint validation (Solovev, 2024). Low reproducibility is alarmingly common: fewer than 20% of reported aging-related multi-omics signatures are replicated in independent cohorts, due to biological (diet, circadian rhythms), technical (platforms, pipelines), and statistical (multiple testing) factors (Skov et al., 2021; Odenkirk et al., 2021). Tissue-specific variability poses a unique barrier. Most studies rely on blood or skin, yet aging signatures in hippocampus, bone marrow, or kidney differ dramatically from peripheral blood, and the lack of multi-tissue data hinders organ-specific interventions (Biga et al., 2025; Long et al., 2025).

Collectively, these limitations—batch effects, population bias, cross-sectional designs, poor reproducibility, and tissue specificity—explain poor clinical translation of multi-omics-derived anti-aging targets. Concerning standardization, variations in detection methods and data analysis pipelines across laboratories further hinder result comparability. For example, batch effects in RNA-seq data from different platforms compromise the reliability of research findings (Skov et al., 2021). In terms of cost, the prohibitive expense of multi-omics assays restricts their application in large-scale populations. Despite significant cost reductions for whole-genome sequencing, it remains above $1000, impeding widespread adoption (Patel et al., 2020). Taken together, insufficient clinical validation of AI-discovered targets, with preclinical promise rarely translating to measurable benefits in humans.

The limitations of AI technology include data bias, poor model interpretability, and insufficient generalization capabilities. Regarding data bias, training data for AI models often originates from specific populations, resulting in performance declines in other groups. For instance, epigenetic clocks trained on European populations show reduced accuracy in Asian populations (Wu et al., 2025; Bajenova et al., 2019). Moreover, the overall size of high-quality labeled aging datasets remains relatively small, restricting model training, overfitting risks, and weakening external validity. A further critical issue is the lack of well-validated, clinically qualified aging biomarkers; most AI-identified signatures are associative rather than causally validated, and few have been verified in large-scale randomized controlled trials. Most prediction models remain “black box” systems with limited causal interpretability, preventing researchers from fully understanding biological mechanisms driving AI predictions and thereby undermining regulatory trust and clinical adoption. Most importantly, AI-discovered targets, senolytics, and predictive models remain severely hampered by insufficient clinical validation, with preclinical promise rarely translating to measurable benefits in humans. In terms of model interpretability, the black-box nature of deep learning models makes their decision-making process difficult to comprehend. WormNet, based on convolutional neural networks, cannot explain why certain image features correlate with lifespan, hampering its clinical application and trust (Galimov and Yakimovich, 2022). Regarding generalization capabilities, AI models exhibit significant performance variations across different datasets. For instance, the DrugPipe platform demonstrates high prediction accuracy for certain targets but performs poorly on others, hindering its broad application (Pham et al., 2025). Collectively, small datasets, inadequate validated biomarkers, black-box model structures, limited causal interpretability, and insufficient clinical validation remain substantial obstacles that continue to impede the successful clinical translation of AI-based approaches in aging research.

### 7.3 Strategies for standardizing multi-omics data

The major barrier to the clinical translation of multi-omics-derived aging biomarkers and therapeutic targets is the substantial heterogeneity across cohorts, analytical platforms, and populations. Beyond recognizing these limitations, future studies should adopt practical methodological frameworks that improve reproducibility, interoperability, and model generalizability.

First, federated learning provides a privacy-preserving solution for multicenter data integration by enabling collaborative model training without sharing individual-level data. Instead of transferring raw datasets, only model parameters are exchanged, allowing robust learning across geographically distributed cohorts while mitigating population bias and improving the generalizability of AI models (Rieke et al., 2020; Xu et al., 2021). Such frameworks are particularly valuable for developing aging biomarkers and biological age clocks applicable across diverse ethnic populations.

Second, harmonized multi-omics analytical pipelines are essential for reducing technical variability. Standardized procedures covering sample collection, sequencing, quality control, normalization, batch-effect correction (e.g., ComBat or Harmony), and statistical modeling should be implemented to improve cross-study comparability and reproducibility (Skov et al., 2021; Odenkirk et al., 2021). Community-driven consensus protocols will provide a solid foundation for multicenter validation and large-scale meta-analyses.

Third, adoption of the FAIR (Findable, Accessible, Interoperable, and Reusable) data principles should become standard practice in aging research. Standardized metadata annotation, public deposition of omics datasets and analytical workflows, and interoperable data formats will facilitate independent validation, secondary analyses, and collaborative model development (Wilkinson et al., 2016; Sansone et al., 2019). Funding agencies and journals should further promote FAIR-compliant data management to enhance transparency and reproducibility.

Collectively, federated learning, harmonized analytical pipelines, and FAIR data principles constitute complementary strategies for overcoming data heterogeneity. Their integration will substantially improve the robustness, reproducibility, and clinical translatability of multi-omics and AI-driven anti-aging drug discovery.

## 8 Conclusion

The integration of multi-omics and artificial intelligence technologies has brought new opportunities to anti-aging drug research, overcoming the limitations of traditional research paradigms. By systematically analyzing the molecular mechanisms of aging, this approach accelerates drug target discovery, drug screening, and clinical translation, providing novel insights and technical foundations for extending human healthspan.

The deep integration of multi-omics and AI has significantly propelled the transition of anti-aging drug research from traditional paradigms to data-driven approaches, demonstrating unique value in target discovery, drug screening, and clinical translation. However, ethical controversies, technical bottlenecks, and regulatory gaps remain major obstacles hindering development in this field. Looking ahead, with the continuous development of multi-omics and AI technologies, ongoing discovery of novel drug targets, interdisciplinary collaboration, gradual adoption of personalized treatment, and continuous improvement in policy regulation, it is expected to achieve precision development and widespread application of anti-aging drugs. This will provide new solutions for alleviating population aging pressures, improving quality of life for the elderly, and extending human healthspan.
